# Prevalence of Hair Loss After COVID-19 Infection in Makkah Region, Saudi Arabia

**DOI:** 10.7759/cureus.29285

**Published:** 2022-09-18

**Authors:** Rahaf A Abdulwahab, Bushra M Aldajani, Nujood K Natto, Azad M Janabi, Orjuwan I Alhijaili, Norah T Faqih, Aymen Alharbi

**Affiliations:** 1 Medicine and Surgery, Umm Al-Qura University, Makkah, SAU; 2 Dermatology & Hair Disorders, King Abdulaziz Hospital, Makkah, SAU

**Keywords:** sars‑cov‑2, alopecia., telogen effluvium, hair loss, covid-19

## Abstract

Introduction

COVID-19, caused by SARS-CoV-2, is a worldwide pandemic with the most main symptoms seeming to be flu-like and fever. Besides that, dermatological manifestations have been reported as extra respiratory symptoms in previous studies. The aim of this study is to measure the prevalence of hair loss after COVID-19 infection in Saudi Arabia, and we hope to improve the knowledge on hair loss - a cause of common dermatological consultation that frequently becomes a stressful event associated with the pandemic - among all the physicians in all the specialties.

Methodology

This was a record-based retrospective cross-sectional, multicenter study conducted in four centers in the Makkah region. We identified 343 patients who visited the dermatology clinic for hair loss during the period 2020 to 2022.

Results

Evidence suggests that hair loss had been the most frequently reported post COVID-19 manifestation (48%). Our results revealed nearly half of the participants (48.5%) noticed hair loss increases by more than 120 hairs per day after COVID-19 infection, as well as half of the participants (52.6%) reported seeing hair accumulation on a pillow. Our results showed that telogen effluvium (TE) was the most reported type (156, 86.7%), followed by alopecia areata (15, 8.3%) and androgenic alopecia (9, 5.0%).

Conclusion

The results suggest that hair loss is noticeably prevalent in patients following COVID-19 infection, especially in females and patients with multiple comorbidities. Telogen effluvium (TE) was the most prevalent type of hair loss recognized among the patients.

## Introduction

The coronavirus disease 2019 (COVID-19) caused by severe acute respiratory syndrome coronavirus 2 (SARS-CoV-2) appeared in the city of Wuhan in China, and it spread around the world. It is transmitted rapidly among humans by Inhalation of respiratory droplets or contact [1,2]. When it comes to SARS-CoV-2 infection, it can present asymptomatically [3] and COVID-19 presentation can range from flu-like illness up to severe complications [4]. Furthermore, epidemiological studies showed that the elderly population was more susceptible to developing severe diseases, while children tend to have milder symptoms. It's currently a global pandemic that's hitting almost all countries. According to the WHO, one out of every 20 people will require intensive care, which may involve being sedated and placed on a ventilator [5]. Moreover, there are multiple variables that contribute to higher morbidity and mortality in COVID-19 infections such as diabetes, obesity, chronic obstructive pulmonary disease (COPD), advanced age, and male sex [6].

The consequences after recovery from COVID-19 infection have been of special concern. Therefore, researchers are investigating the phases beyond COVID-19 infection and have defined it as post-acute COVID-19 syndrome, which is characterized by persistent symptoms and/or delayed or long-term complications of SARS-CoV-2 infection beyond four weeks from the onset of symptoms that affect multiple organs. Here, we will focus on dermatological sequelae specifically hair loss [7]. Regarding the duration of the post-acute COVID-19 sequelae, it lasts for four weeks in a confirmed case. The two phases of a post-acute COVID-19 syndrome are: first, the subacute phase that includes symptoms and abnormalities present from one to three months after acute COVID-19; second, chronic or post-COVID-19 syndrome, which includes symptoms and abnormalities persisting or present after three months of the onset of acute COVID-19 and not attributable to alternative diagnoses [7].

In view of dermatological sequelae specifically hair loss, it is also described in the literature as telogen effluvium (TE). It occurs post COVID-19 infection by two mechanisms [8]. The first one is by direct damage of endothelial cells of small vessels by the virus, second is by indirect damage by the release of cytokines and development of immune inflammation around hair follicles and activation of coagulation cascade which leads to thrombus formation in the vessels that supply the hair follicles resulting in ischemia and necrosis of follicles [8]. Also, some medications such as antibiotics and anticoagulants can lead to TE [8].

The aim of this study is to measure the prevalence of hair loss after COVID-19 infection in Makkah region, Saudi Arabia, and we hope to improve the knowledge on hair loss - a cause of common dermatological consultation that frequently becomes a stressful event associated with the pandemic - among all the physicians in all the specialties.

## Materials and methods

Study design and patients’ records

This was a multi-center, record-based retrospective cross-sectional study conducted in four centers in the Makkah region including King Abdulaziz Hospital, King Faisal Hospital, Heraa General Hospital, and Al-Noor Specialist Hospital. The study was conducted between January 2021 and March 2022. We identified 343 patients who visited the dermatology clinic for hair loss during the period 2020 to 2022. We enrolled patients of all age groups and only confirmed COVID-19 cases of both genders. On the other hand, we excluded 17 patients with iron deficiency anemia (IDA), post-partum female patients, and patients with a history of hair disorders.

Study database

Data for the analysis were obtained from the patient records who visited the dermatology clinic. The patient records contain sociodemographic information, past medical history of chronic disease, history of hair disorders before COVID-19, COVID-19 detection and history of hospitalization and medication used, and laboratory data for COVID-19 detection. Approval was obtained from the Institutional Review Board office of the Ministry of Health, Makkah (IRB Number: H-02-K-076-1021-580).

Definition of the confirmed cases of COVID-19 from a laboratory perspective

COVID-19 infection was defined according to the newly published paper as an integration of clinical characteristics, symptoms, and laboratory results. Regarding the new evidence of confirming COVID-19 cases, there were two tests: first, etiological diagnosis that may be direct, identifying genetic material of SARS-CoV-2 by real-time polymerase chain reaction (RT-PCR); second was indirect test for determining the humoral immune response to SARS-CoV-2 by different serological tests [9].

Data analysis

After data were extracted, it was revised, coded, and fed to statistical software Statistical Package for the Social Sciences (SPSS) version 22 (IBM Corp., Armonk, NY). All statistical analysis was done using two-tailed tests. p-value less than 0.05 was statistically significant. Descriptive analysis based on frequency and percent distribution was done for all variables including patients’ personal data, BMI, smoking, co-morbidities and family history of alopecia. Also, patients' COVID-19 infection data were tabulated besides post-infection hair loss and alopecia-related frequency, types and duration. Crosstabulation was used to assess factors associated with post COVID-19 infection hair loss among study patients. Relations were tested using Pearson's chi-squared test and exact probability test for small frequency distributions.

## Results

A total of 343 patients were enrolled in the study using a data collection sheet. Patients' ages ranged from 11 to 70 years with a mean age of 29.5 ± 10.6 years. A total of 261 (76.3%) patients were females and 180 (52.6%) patients were single while 135 (39.5%) patients were married. Smoking was reported among 70 (20.5%) patients. A total of 112 (32.75) patients were overweight while 46 (13.5%) patients were obese. Regarding chronic diseases, 20 (5.8%) patients were diabetic, 15 (4.4%) patients had hypertension while 288 (84.2%) patients had no chronic health problem. A total of 50 (14.6%) patients had a family history of androgenic alopecia or alopecia areata (Table 1).

**Table 1:** Bio-demographic data of COVID-19 study patients, Makkah Region, Saudi Arabia

Bio-demographic data	No	%
Age in years		
<20	41	12.0%
20-29	167	48.8%
30-39	64	18.7%
40+	70	20.5%
Gender		
Male	81	23.7%
Female	261	76.3%
Marital status		
Single	180	52.6%
Married	135	39.5%
Divorced / widow	27	7.9%
Smoking		
Yes	70	20.5%
No	272	79.5%
BMI		
Normal	184	53.8%
Overweight	112	32.7%
Obese	46	13.5%
Chronic diseases		
No chronic disease	288	84.2%
Hypertension	20	5.8%
Diabetes mellitus	15	4.4%
Respiratory disease	6	1.8%
Hypothyroidism	5	1.5%
Polycystic ovary syndrome	2	.6%
Cardiovascular disease	2	.6%
Hypersensitivity reaction	1	.3%
Irritable bowel syndrome	1	.3%
Multiple sclerosis	1	.3%
Visual disease	1	.3%
Family history of androgenic alopecia or alopecia areata?		
Yes	50	14.6%
No	292	85.4%

Table 2 shows the COVID-19 infection data among study patients in the Makkah region, Saudi Arabia. A total of 303 (88.6%) patients were diagnosed with COVID-19 infection by nasopharyngeal swab or oral swab, 21 (6.1%) patients were diagnosed by PCR while 18 (5.4%) patients were diagnosed by clinical signs and symptoms. Ten (2.9%) patients were hospitalized, six (60%) patients were in the general ward, while four patients (40%) were in the observation room. Nine (90%) hospitalized patients stayed for less than one week. Also, 10 (2.9%) patients used medications for COVID-19 infection.

**Table 2:** COVID-19 infection data among study patients, Makkah region, Saudi Arabia

COVID-19 infection data	No.	%
Method of COVID-19 diagnosis		
Nasopharyngeal swab	303	88.6%
PCR	21	6.1%
Signs and symptoms of COVID-19	18	5.4
Need for hospitalization		
Yes	10	2.9%
No	332	97.1%
If yes, place of admission		
To general ward	6	60.0%
To observational room	4	40.0%
Duration of hospitalization		
1-2 weeks	1	10.0%
Less than 1 week	9	90.0%
Medication use (anticoagulant, Antibiotics) during COVID-19 infection?		
Yes	10	2.9%
No	332	97.1%

Table 3 shows hair loss and its relation to COVID-19 infection among study patients in the Makkah region, Saudi Arabia. A total of 53 (15.5%) patients had a history of hair loss before COVID-19 infection - 37 (69.8%) patients had telogen effluvium, 11 (20.8%) patients had androgenic alopecia, and five (9.4%) patients had alopecia areata. A total of 166 (48.5%) patients reported that hair loss increased after COVID-19 by more than 120 hairs per day and 180 (52.6%) patients noticed hair collection on a pillow or during a shower. The most reported types were telogen effluvium (86.7%), followed by alopecia areata (8.3%), and androgenic alopecia (5%). Hair loss occurred after 2-3 months of COVID-19 infection among 114 (63.3%) cases, while 43 (23.9%) patients had hair loss after six months of the infection and 23 (12.8%) patients had hair loss immediately after the infection.

**Table 3:** Hair loss and its relation to COVID-19 infection among study patients, Makkah region, Saudi Arabia

Hair loss data	No.	%
History of hair loss before COVID-19		
Yes	53	15.5%
No	289	84.5%
If yes, type of hair loss do the patients have		
Alopecia areata	5	9.4%
Androgenic alopecia	11	20.8%
Telogen effluvium	37	69.8%
Do you think your hair loss increased after COVID-19 by more than 120 hairs per day?		
Yes	166	48.5%
No	176	51.5%
Did you notice hair collection on a pillow?		
Yes	180	52.6%
No	162	47.4%
Type of hair loss after COVID-19 infection		
Alopecia areata	15	8.3%
Androgenic alopecia	9	5.0%
Telogen effluvium	156	86.7%
Time of occurrence of hair loss after infection		
After 2-3 months	114	63.3%
After 6 months and more	43	23.9%
After COVID-19 immediately	23	12.8%

Table 4 shows factors associated with post COVID-19 infection hair loss among study patients in the Makkah region, Saudi Arabia. Post-infection hair loss was reported among 60.2% of female patients compared to 28.4% of male patients with recorded statistical significance (P=.001). Also, 61.1% of patients with chronic health problems had post COVID-19 infection hair loss versus 51% of others without (P=.049). Other factors were insignificantly associated with post-infection hair loss.

**Table 4:** Factors associated with post COVID-19 infection hair loss among study patients, Makkah region, Saudi Arabia P: Pearson X^2^ test $: Exact probability test * P < 0.05 (significant)

Factors	Did you notice hair collection on a pillow or during a shower?				p-value
	Yes		No		
	No.	%	No.	%	
Age in years					.944
< 20	20	48.8%	21	51.2%	
20-29	89	53.3%	78	46.7%	
30-39	33	51.6%	31	48.4%	
40+	38	54.3%	32	45.7%	
Gender					.001*
Male	23	28.4%	58	71.6%	
Female	157	60.2%	104	39.8%	
Marital status					.590
Single	90	50.0%	90	50.0%	
Married	75	55.6%	60	44.4%	
Divorced / widow	15	55.6%	12	44.4%	
Smoking					.621
Yes	35	50.0%	35	50.0%	
No	145	53.3%	127	46.7%	
BMI					.319
Normal	90	48.9%	94	51.1%	
Overweight	63	56.3%	49	43.8%	
Obese	27	58.7%	19	41.3%	
Chronic diseases					.049*
Yes	33	61.1%	21	38.9%	
No	147	51.0%	141	49.0%	
Family history of androgenic alopecia or alopecia areata					.687
Yes	25	50.0%	25	50.0%	
No	155	53.1%	137	46.9%	
Hospitalization due to COVID-19					.417^$^
Yes	4	40.0%	6	60.0%	
No	176	53.0%	156	47.0%	

## Discussion

The current study was conducted to assess the prevalence of hair loss after COVID-19 infection in the Makkah region and the factors associated with post COVID-19 hair loss among study patients. In the present study, only confirmed COVID-19 cases were analyzed (Table 2). There is increasing evidence suggesting that hair loss had been the most frequently reported post COVID-19 manifestation (2,800, 48%), from a total of 5,264 (89.4%) participants who were treated at home, 396 (6.7%) who were treated in a hospital, and 231 (3.9%) who were treated in an intensive care unit, according to a study conducted in Brazil [10]. In addition, a systematic review and meta-analysis aimed to assess the prevalence of long-term effects of COVID-19 on hair loss among 55 patients, and it was estimated to be one of the most common side effects of COVID-19 infection, with a prevalence of 25% [11], compared to our results that revealed nearly half of the participants (48.5%) noticed hair loss increases by more than 120 hairs per day after COVID-19 infection, as well as half of the participants (52.6%) reported seeing hair accumulation on a pillow (Table 3). The significance of cutaneous involvement in COVID-19 was not recognized early in the pandemic but was discovered much later and was critical because it was a presenting complaint in some patients [12]. Müller-Ramos et al. discovered that telogen effluvium (TE) is the main cause of hair shedding after COVID-19 as a cutaneous involvement manifestation, and it has been reported in six previous studies, but because patients were not examined by a dermatologist, it was not possible to rule out other diagnoses (e.g., alopecia areata) [10].

Consequently, a study conducted in Turkey documented the increase in the prevalence of TE during the pandemic, and it was found in 27.9% of the 563 participants [13]. Moreover, according to Singh et al.'s systematic review, 25% of the 465 patients reported hair loss after recovering from COVID-19 and were later diagnosed with acute TE [14], that was in accordance with our results that showed telogen effluvium (TE) was the most reported type (156, 86.7%), followed by alopecia areata (15, 8.3%) and androgenic alopecia (9, 5.0%) (Table 3). Furthermore, in the present study, we found that 114 (63.3%) of patients experienced hair loss after 2-3 months of COVID-19 infection, whereas 43 (23.9%) experienced hair loss after six months of infection and 23 (12.8%) experienced hair loss immediately after infection (Table 3). And this is similarly supported by a cross-sectional study that had been done among 39 patients with post COVID-19 hair loss confirmed by a hair pull test and they all reported severe hair loss within 2-3 months after infection [15].

Regarding the factors associated with post COVID-19 infection hair loss that has been reported in a multicenter study that shows the female gender, long hospital stays, a higher number of comorbidities, and a higher number of symptoms at hospital admission were risk factors associated with a higher number of post COVID-19 hair loss [16]. In the present study, most of the COVID-19 patients were female (261, 76.3%) (Table 1), and there were different chronic diseases namely hypertension (20, 5.8%), diabetes mellitus (15, 4.4%), respiratory disease (6, 1.8%), hypothyroidism (5, 1.5%), polycystic ovary syndrome (PCOS) (2, 0.6%), cardiovascular disease (2, 0.6%) and other reported chronic disease (Table 1). Similarly, the adjusted analysis revealed a significant value of the female gender factor (157, 60.2%) highly associated with the occurrence of hair loss on a pillow or during a shower after COVID-19 infection with (P = .001). Additionally, the presence of chronic disease (33, 61.1%) is a significant factor that plays a role in developing hair loss after COVID-19 infection with P = .049 (Table 4). In an opposing view, the hospitalization due to COVID-19 infection mainly determines the severity of the COVID-19 infection, and it shows insignificant value regarding the factors associated with post COVID-19 infection hair loss with P = .417% means the severity of COVID-19 infection doesn't play a significant role in developing a hair loss (Table 4). However, we couldn't assess the severity of COVID-19 based on the hospital admission only, as it may require other clinical, radiological, and laboratory data that mainly determine the severity of the COVID-19 infection cases.

As a result of that, one of the recognized limitations of our study is it didn't collect objective measures of an admitted patient with COVID-19 such as inflammatory biomarkers, and blood oxygen saturation which mainly determines the severity of cases as well as the severity of development of cytokine storm and inflammatory response that is known mechanism to affect all the body cells in the form of post COVID-19 infection sequela. There are other limitations regarding the pictures of patients with hair loss that couldn't be taken to preserve the privacy of the enrolled patients.

## Conclusions

COVID-19 infection is now a frequent and common cause of hair loss. Telogen effluvium (TE) is noticeably prevalent in patients following COVID-19 infection, especially in females and patients with multiple comorbidities. TE was the most prevalent type of hair loss recognized among the patients, and it's expected to occur 2-3 months post-infection. TE would be one of the important aspects of the long-term COVID-19 sequela, which will impact the quality of life of the affected person. As a result of that, we hope to improve the knowledge on hair loss - a cause of common dermatological consultation that frequently becomes a stressful event associated with the pandemic - among all the physicians in all specialties.
